# Green tea polyphenols supplementation and Tai Chi exercise for postmenopausal osteopenic women: safety and quality of life report

**DOI:** 10.1186/1472-6882-10-76

**Published:** 2010-12-09

**Authors:** Chwan-Li Shen, Ming-Chien Chyu, Barbara C Pence, James K Yeh, Yan Zhang, Carol K Felton, Susan Doctolero, Jia-Sheng Wang

**Affiliations:** 1 Department of Pathology, Texas Tech University Health Sciences Center, Lubbock, Texas, USA; 2 Laura W. Bush Institute for Women's Health, Texas Tech University Health Sciences Center, Lubbock, Texas, USA; 3 Department of Laboratory Science and Primary Care, Texas Tech University Health Sciences Center, Lubbock, Texas, USA; 4 Department of Mechanical Engineering, Texas Tech University, Lubbock, Texas, USA; 5 Department of Health, Exercise, and Sport Sciences, Texas Tech University, Lubbock, Texas, USA; 6 Graduate Healthcare Engineering Option, Texas Tech University, Lubbock, Texas, USA; 7 Applied Bench Core Laboratory, Winthrop-University Hospital, Mineola, New York, USA; 8 Department of Family and Community Medicine, Texas Tech University Health Sciences Center, Lubbock, Texas, USA; 9 Department of Obstetrics and Gynecology, Texas Tech University Health Sciences Center, Lubbock, Texas, USA; 10 Clinical Research Center, Texas Tech University Health Sciences Center, Lubbock, Texas, USA; 11 Department of Environmental Health Science, University of Georgia, Athens, Georgia, USA

## Abstract

**Background:**

Evidence suggests that both green tea polyphenols (GTP) and Tai Chi (TC) exercise may benefit bone health in osteopenic women. However, their safety in this population has never been systematically investigated. In particular, there have been hepatotoxicity concerns related to green tea extract. This study was to evaluate the safety of 24 weeks of GTP supplementation combined with TC exercise in postmenopausal osteopenic women, along with effects on quality of life in this population.

**Methods:**

171 postmenopausal women with osteopenia were randomly assigned to 4 treatment arms for 24 weeks: (1) Placebo (500 mg starch/day), (2) GTP (500 mg GTP/day), (3) Placebo + TC (placebo plus TC training at 60 min/session, 3 sessions/week), and (4) GTP + TC (GTP plus TC training). Safety was examined by assessing liver enzymes (aspartate aminotransferase, alanine aminotransferase), alkaline phosphatase, and total bilirubin at baseline and every 4 weeks. Kidney function (urea nitrogen and creatinine), calcium, and inorganic phosphorus were also assessed at the same times. Qualify of life using SF-36 questionnaire was evaluated at baseline, 12, and 24 weeks. A mixed model of repeated measures ANOVA was applied for analysis.

**Results:**

150 subjects completed the study (12% attrition rate). The compliance rates for study agents and TC exercise were 89% and 83%, respectively. Neither GTP supplementation nor TC exercise affected liver or kidney function parameters throughout the study. No adverse event due to study treatment was reported by the participants. TC exercise significantly improved the scores for role-emotional and mental health of subjects, while no effect on quality of life was observed due to GTP supplementation.

**Conclusions:**

GTP at a dose of 500 mg/day and/or TC exercise at 3 hr/week for 24 weeks appear to be safe in postmenopausal osteopenic women, particularly in terms of liver and kidney functions. TC exercise for 24 weeks (3 hr/wk) significantly improved quality of life in terms of role-emotional and mental health in these subjects. ClinicalTrials.gov identifier: NCT00625391.

## Background

In a recent comprehensive review [1], we suggest that tea and its bioactive components might reduce bone fracture risk by benefiting bone mineral density (BMD) and supporting osteoblastic activities while suppressing osteoclaistic activities, possibly due to their antioxidant and/or anti-inflammatory functions. Among different types of tea, green tea polyphenols (GTP, extract of green tea) has shown its osteo-protective effects by decreasing oxidative stress [2,3], increasing activity of antioxidant enzymes [2], and decreasing expression of proinflammatory mediators in rodent models [3]. However, limited information is available on the protective effect of consumption of tea or its bioactive components (e.g., GTP) on bone health in postmenopausal women. On the other hand, Tai Chi (TC), a form of mind-body, moderate-intensity, aerobic and muscular fitness exercise, has also shown to potentially benefit bone health [4-7]. However, there is limited information based on systematic study of TC's effect on bone health in postmenopausal women with low bone mass. Therefore, the long-term goal of the study is to investigate the effect of GTP and TC exercise on bone health in the targeted population. This paper focuses on the safety and impact on quality of life associated with this combined intervention. Results of bone, inflammation and oxidative stress parameters will be reported in a separate paper.

Legislation in use of complementary and alternative medicine (i.e., herbal/dietary supplement) is not uniform, even lacking in many countries. In the US, green tea extract is labeled as a dietary supplement which does not seem to require pre-clinical tests, and its traditional use proves not to be harmful in the specified condition of use [8]. Although green tea has been a popular beverage for centuries, a systemic review by the recent United States Pharmacopeia (USP) of 216 case reports on green tea products revealed 34 reports concerning liver damage [9]. Among them, 27 reports were categorized as possible causality and 7 reports as probable causality. Based on this review, the USP Dietary Supplement Information Expert Committee determined that when dietary supplement products containing green tea extract are used and formulated appropriately, the Committee is unaware of significant safety issues that would prohibit monograph development. A caution statement needs to be included in the labeling section [9].

On the other hand, based on published hepatotoxicity episodes, Mazzanti et al. [10] concluded that there can be no longer a reasonable doubt that ingestion of concentrated extracts of green tea and infusions of green tea itself poses a real and growing risk to liver health. The hepatotoxicity is probably due to (-)-epigallo-catechin gallate or its metabolites which, under particular conditions related to the patient's metabolism, can induce oxidative stress in the liver. In a few cases, toxicity related to concomitant medications could also be involved [10].

The above evidence suggests that it is important to assess safety issues in conducting a long-term clinical study involving green tea extract as a treatment. However, most of the published green tea clinical studies were either short-term (≤ 12 weeks) [11-13], with a longer study period but little or limited information on safety data related to liver function [14,15], or relatively small sample sizes [11-15]. The detailed safety information is important because for all of the interest in clinical studies using green tea as study agents, lacking such information hinders the research development. The present work is the first GTP safety report on liver and kidney functions based on a larger sample size in a 24-week placebo-controlled and randomized clinical trial.

Tai Chi has been investigated in many clinical studies, and is generally considered a safe intervention/treatment in population with various health issues [16]. However, no study evaluated the effect of TC in conjunction with GTP supplementation on liver and kidney function in any study population. It is not clear if Tai Chi exercise would interact with GTP to attenuate green tea related toxicity in our study subjects. Such safety data are important to future clinical studies using GTP and/or Tai Chi as study treatment.

Therefore, the objective of this paper is to evaluate the safety of 24 weeks of GTP supplementation combined with TC exercise in postmenopausal osteopenic women. In addition to safety, the effects of treatment arms on quality of life (as assessed by SF-36 questionnaires) are also reported.

## Methods

### Study participants

Postmenopausal women were recruited primarily through flyers, local TV, radios, newspaper, municipal community centers and clinics to participate in this study. The complete study protocol has been reported in detail previously [17] and only a brief description is provided here.

Inclusion criteria were (i) postmenopausal women (at least 2 years after menopause) with osteopenia (mean lumbar spine and/or hip bone mineral density (BMD) T-score between 1 and 2.5 standard deviation (SD) below the young normal sex-matched areal BMD of the reference database) [12], (ii) normal function of thyroid (thyroid-stimulating hormone (TSH) > 0.3 and < 5.0 mU/L), liver (bilirubin ≤ 2.0 mg/dL, aspartate aminotransferase (AST)/alanine aminotransferase (ALT) < 3 × upper limit of normal), and kidney (serum creatinine (Crt) ≤ 2.0 mg/dL, blood urea nitrogen (BUN) < 1.5 times), (iii) serum alkaline phosphatase (ALP) (33 - 130 U/L), calcium (Ca) (8.6 - 10.2 mg/dL), and inorganic phosphorus (Pi) (2.5 - 4.5 mg/dL) were within normal ranges, (iv) and serum 25-hydroxy vitamin D (25(OH)D) ≥ 20 ng/mL.

Women were excluded if they (i) had a disease condition or were on medication known to affect bone metabolism, (ii) had a history of cancer except for treated superficial basal or squamous cell carcinoma of the skin, (iii) had uncontrolled intercurrent illness or physical condition that would be a contraindication to exercise, (iv) had depression, cognitive impairment, or (v) were unwilling to accept randomization. Written informed consent was obtained from all the participants before enrollment. The study was approved by the Texas Tech University Health Sciences Center Institutional Review Board.

### Study design and intervention

This was a 24-week, placebo-controlled, randomized intervention trial to investigate the effects of GTP and TC on bone parameters. Participants were randomly assigned to one of the four treatment groups:

▪ Placebo group: medicinal starch 500 mg daily

▪ GTP group: GTP 500 mg daily

▪ Placebo + TC group: medicinal starch 500 mg daily and 24-move simplified Yang-style TC training (60 minutes per session, 3 sessions per week)

▪ GTP + TC group: GTP 500 mg daily and 24-move simplified Yang-style TC training (60 minutes per session, 3 sessions per week)

Medicinal starch and GTP study agents were supplied by Zhejiang Yuxin Pharmaceutical Co., Ltd., China (US FDA IND number 77,470). The main GTP components were 99.25% pure, with 46.5% of epigallocatechin-3-gallate (EGCG), 21.25% of epigallocatechin (ECG), 10% of epicatechin (EC), 7.5% of epicatechin-3-gallate (EGC), 9.5% of gallocatechin gallate (GCG), and 4.5% of catechin. The daily dose of GTP or placebo material was divided into two capsules (250 mg each). During the 24-week intervention, all participants were provided with 500 mg elemental calcium and 200 IU vitamin D (as cholecalciferol) daily.

### Randomization and blinding

To ensure comparable distribution across treatment arms, eligible participants were stratified before randomization by a fixed randomized scheme based on age (≥ 65 or < 65 years old), history of green tea consumption, and history of mind-body exercise. Both the study participants and investigators responsible for the day-to-day operation and data analyses were blinded to the GTP/placebo group status.

### Measurements

Medical history, physical activity level, depression (mood), and cognitive impairment assessment were collected at the time of enrollment. The depression (mood) assessment was measured by the Yesavage self-rated Geriatric Depression Score [18]. BMD was determined at baseline for the screening purpose by dual energy X-ray absorptiometry (DEXA) (Norland Excel X-Ray Bone Densitometer). Also at baseline for screening purposes only, overnight fasting blood and urine samples were collected for the measurement of concentrations of serum 25(OH)D and TSH by a certified diagnostic laboratory (Quest Diagnostics, Dallas, TX).

Laboratory blood chemistry parameters, including ALP, BUN, bilirubin (Bil), AST, ALT, Ca, Pi, and Crt were assessed in overnight fasting blood samples taken at baseline and every 4 weeks throughout the study period. All samples were processed and analyzed in a certified diagnostic laboratory (Quest Diagnostic Laboratory, Dallas, TX).

General health status was measured with the Medical Outcomes Study 36-item short form Health Survey (SF-36, version 2) at baseline, 12 and 24 weeks of study. SF-36 has been reported to have good validity, internal consistency, and reliability in the assessment of physical and mental health status of subjects and their progression [19,20]. The SF-36 consists of eight dimensions of health (physical function, bodily pain, general health, vitality, mental health, social function, and role of physical and emotional health) in the conduct of daily activity [21].

### Adverse event monitoring

In the course of the 24-week clinical trial, adverse events associated with study agents were self-reported by the participants, and by monitoring liver enzyme activities, AST and ALT in particular, through blood analysis. Participants in the TC exercise groups (placebo + TC and the GTP + TC groups) were also queried about any adverse events due to TC during TC training sessions. They were also encouraged to self-report any adverse events by telephone. All observed and self-reported adverse events, regardless of suspected causal relationship to the study treatments, were recorded on the adverse event form throughout the study.

### Compliance

Adherence/compliance of GTP or placebo study agents was determined as the percentage of all capsules of GTP or placebo capsules ingested throughout the study period. Compliance of TC classes was assessed by TC class attendance record for each TC session.

### Statistical analysis

For this longitudinal study, a model of repeated measurements with random effect error terms was used with "intention-to-treat analysis" for missing data, if applicable. Statistical software SPSS 16.0 (Clicago, IL, USA) was employed to conduct the analyses, controlling for the within subject correlation. First, participant characteristics were compared to detect any difference among the four groups at baseline. Second, changes in the measurements between baseline and the follow-ups were analyzed. For between-group differences over time, a repeated measure ANOVA was conducted and controlled for within-subject correlation. The two treatment factors are GTP (vs. placebo) and TC (vs. no TC). Third, the characteristics of participants who dropped out were compared with those of the participants who stayed for the entire study period in order to detect potential biases.

## Results

### Participants

**Figure 1:**
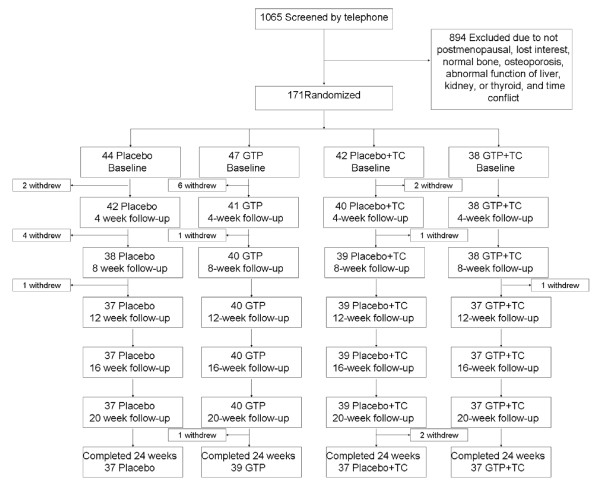
**Study flow chart**.

A total of 1065 patients were prescreened. Among them, 171 were qualified and randomized, and 150 completed the 24-week study (Figure 1). Seven (16%) participants in the Placebo arm, 8 (17%) in the GTP arm, 5 (12%) in the Placebo + TC arm, and 1 (3%) in the GTP + TC arm withdrew before the end of the study, due to accidental fall (1 subject), relocation (2 subjects), time conflicts (6 subjects), lost to follow-up (5 subjects), and lost interest (7 subjects). Baseline characteristics were similar among different treatment groups (Table 1). No statistically significant differences between the subjects who withdrew from the study and those who completed the study were observed in any parameter listed in Table 1. All subjects were instructed to maintain their pre-existing physical activity, dietary habits, and medications, if any, throughout the study. Based on the results of pill count, the compliance rate was 89% for both GTP and placebo capsules. The compliance rate for TC classes was 83%.

**Table 1:** Baseline demographic characteristics of study population

Variables	Placebo	GTP	Placebo+TC	GTP+TC	*P *value
***Number***	44	47	42	38	
					
***Age ****[y], mean ± SD*	57.6 ± 7.5	56.5 ± 5.5	58.3 ± 7.7	57.6 ± 6.7	NS
***Years after menopause***	12.5 ± 8.4	11.2 ± 8.3	14.2 ± 11.1	11.4 ± 9.1	NS
***Height ****[cm]*	162.8 ± 7.8	162.7 ± 5.4	161.9 ± 6.2	164.1 ± 7.1	NS
***Weight ****[kg]*	77.4 ± 15.4	74.5 ± 11.9	74.1 ± 12.1	72.9 ± 14.6	NS
***Body mass index ****[kg/m^2^]*	29.2 ± 6.1	28.1 ± 4.4	28.3 ± 4.9	27.1 ± 5.8	NS
					
***Bone mineral density ****[T-score]*					
*Femoral Neck*	-1.50 ± 0.62	-1.51 ± 0.64	-1.64 ± 0.56	-1.69 ± 0.59	NS
*Trochanter*	-1.05 ± 0.81	-1.10 ± 0.70	-1.27 ± 0.62	-1.29 ± 0.71	NS
*Total spine*	-0.74 ± 0.56	-0.75 ± 0.67	-0.95 ± 0.65	-0.97 ± 0.51	NS
*L1-L4*	-0.60 ± 0.85	-0.53 ± 1.09	-0.65 ± 0.76	-0.75 ± 0.83	NS
					
***Serum 25 (OH)D ****[ng/mL]*	32.5 ± 8.4	36.2 ± 11.0	31.5 ± 11.5	30.1 ± 6.6	NS
					
***Serum PTH ****[pg/mL]*	47.8 ± 20.1	45.6 ± 22.5	48.2 ± 22.3	47.2 ± 27.1	NS
					
***Serum TSH ****[mIU/L]*	2.50 ± 1.38	2.09 ± 1.13	2.38 ± 0.94	2.56 ± 1.30	NS
					
***General health questions ****[n (%)]*					
General health rated "good"	35 (79.5)	38 (80.9)	38 (90.4)	30 (78.9)	NS
Height decrease with age	3 (6.8)	10 (21.2)	7 (16.6)	9 (23.6)	NS
Broken bone as adult	9 (20.4)	13 (27.6)	7 (16.6)	14 (36.8)	NS
History of osteopenia	9 (20.4)	8 (17.0)	9 (21.4)	9 (23.9)	NS
Family history of low bone mass	19 (36.3)	24 (51.0)	13(30.9)	23 (60.5)	NS
History of osteoarthritis	9 (20.4)	5 (10.6)	4 (9.5)	4 (10.5)	NS
Severe joint or muscle pain	16 (36.3)	12 (25.5)	9 (21.4)	9 (23.6)	NS
Back or leg pain	11 (25.0)	12 (25.5)	4 (9.5)	5 (13.1)	NS
History of diabetes	1 (2.3)	5 (10.6)	6 (14.2)	3 (7.8)	NS
History of hypertension	13 (29.5)	9 (19.1)	10 (23.8)	8 (21.0)	NS
					
***Physical activity profiles****, mean *± *SD*					
Exercise frequency [sessions/week]	2.4 ± 2.1	2.0 ± 2.1	1.8 ± 2.0	1.9 ± 2.0	NS
Exercise time [min/session]	22 ± 19	23 ± 21	29 ± 46	23 ± 22	NS
					
***Mood assessment***^§^,* mean *± *SD*	5.7 ± 5.2	5.9 ± 4.9	5.9 ± 5.6	6.3 ± 4.2	NS
					
***Lifestyle profiles ****[n (%)]*					
History of steroid use	9 (20.4)	7 (14.8)	8 (19.0)	12 (31.5)	NS
Antidepressant medication use	20 (40.5)	19 (40.4)	17 (40.4)	18 (47.3)	NS
Estrogen/HRT use	9 (20.4)	10 (21.2)	10 (23.8)	7 (18.4)	NS
Calcium/Vitamin D use	22 (50)	23 (48.9)	21 (50)	17 (44.7)	NS
Trouble sleeping	16 (36.4)	17 (36.1)	16 (38.1)	12 (31.5)	NS
Current cigarette smoking	2 (4.5)	2 (4.2)	2 (4.7)	1 (2.6)	NS
Alcohol consumption	23 (52.2)	18 (38.2)	18 (42.8)	21 (55.2)	NS
Tea consumption	26 (59.1)	23 (48.9)	22 (52.3)	27 (71.0)	NS
Coffee consumption	28 (63.6)	34 (72.3)	28 (66.6)	24 (65.5)	NS
Soda consumption	28 (63.6)	29 (65.9)	23 (54.7)	27 (71.1)	NS
Juice consumption	12 (27.2)	22 (46.8)	19 (45.2)	21 (55.2)	NS

GTP, green tea polyphenols; TC, Tai Chi; 25(OH)D, 25-hydroxy-vitamin D; SD, standard deviation; PTH, parathyroid hormone; TSH, thyroid stimulating hormone; HRT, hormone replacement treatment; NS, not significant (P > 0.005).

^§^Mood assessment was performed by the Yesavage self-rated Geriatric Depression Score.

### Safety

At the baseline, there was no significant difference in any of the blood chemistry parameters among all treatment groups (Table 2). Based on the results of ANOVA, the levels of serum AST and ALT (indicators of liver functions) were not affected by either GTP or TC intervention during the 24-week study period (Table 2). Similarly, neither GTP supplementation nor TC exercise influenced serum BUN in subjects (Table 2). On the other hand, throughout the course of the 24-week intervention, there were significant decreasing trends in levels of serum Bil, ALP, Crt, Ca, and Pi over time with different magnitude in each treatment arm. However, in analyzing interaction between the time factor and the two treatment factors (GTP and TC), these parameters in the subjects were not statistically different over time across all the treatment arms (Table 2).

**Table 2:** Effect of green tea polyphenols supplementation and Tai Chi exercise on blood chemistry in postmenopausal osteopenic women

	Treatment groups				*P *(ANOVA)			
		
Variables	Placebo	GTP	Placebo+TC	GTP+TC	Time	Time × GTP	Time × TC	Time × GTP × TC
**Serum AST**, U/L (reference range: 10-35 U/L)					0.431	0.678	0.599	0.437
*Baseline*	19.8 ± 4.3	21.2 ± 6.3	21.2 ± 5.9	19.3 ± 4.3				
*4 week*	19.8 ± 4.6	20.9 ± 4.8	21.4 ± 6.1	19.8 ± 4.5				
*8 week*	20.5 ± 5.7	22.1 ± 6.7	21.4 ± 5.8	20.8 ± 5.8				
*12 week*	19.9 ± 5.1	20.9 ± 5.8	22.1 ± 7.3	20.4 ± 5.1				
*16 week*	19.8 ± 4.4	22.6 ± 7.4	20.9 ± 6.9	20.3 ± 5.1				
*20 week*	19.3 ± 3.7	21.8 ± 6.9	22.7 ± 12.3	19.3 ± 3.5				
*24 week*	19.3 ± 4.2	20.2 ± 6.2	21.1 ± 7.2	20.3 ± 6.1				
								
**Serum ALT**, U/L (reference range: 6-60 U/L)					0.454	0.955	0.893	0.235
*Baseline*	16.9 ± 5.5	20.4 ± 10.1	19.0 ± 7.0	18.0 ± 6.9				
*4 week*	16.9 ± 4.8	19.2 ± 7.9	19.5 ± 6.6	18.5 ± 7.2				
*8 week*	17.4 ± 7.7	21.2 ± 10.9	20.4 ± 7.8	19.2 ± 9.9				
*12 week*	17.1 ± 6.4	19.9 ± 8.2	19.2 ± 9.0	19.9 ± 9.1				
*16 week*	17.1 ± 5.8	21.8 ± 11.7	19.7 ± 6.9	19.0 ± 8.1				
*20 week*	16.6 ± 5.2	21.6 ± 11.3	20.3 ± 9.4	18.1 ± 5.0				
*24 week*	16.9 ± 6.3	19.1 ± 9.9	18.5 ± 5.7	19.5 ± 10.2				
								
**Serum Bil**, mg/dL (reference: 0.2-1.2 mg/dL)					0.001	0.799	0.493	0.060
*Baseline*	0.58 ± 0.19	0.55 ± 0.19	0.65 ± 0.25	0.64 ± 0.26				
*4 week*	0.58 ± 0.23	0.59 ± 0.23	0.67 ± 0.27	0.63 ± 0.22				
*8 week*	0.57 ± 0.17	0.57 ± 0.21	0.68 ± 0.28	0.59 ± 0.25				
*12 week*	0.58 ± 0.16	0.53 ± 0.22	0.59 ± 0.30	0.60 ± 0.31				
*16 week*	0.58 ± 0.18	0.54 ± 0.18	0.62 ± 0.23	0.55 ± 0.22				
*20 week*	0.56 ± 0.21	0.56 ± 0.23	0.64 ± 0.28	0.57 ± 0.21				
*24 week*	0.56 ± 0.20	0.54 ± 0.20	0.60 ± 0.17	0.56 ± 0.23				
								
**Serum ALP**, U/L (reference range: 33-130 U/L)					< 0.001	0.925	0.149	0.438
*Baseline*	75.3 ± 18.6	84.1 ± 19.9	81.6 ± 20.1	86.8 ± 25.2				
*4 week*	77.1 ± 17.7	85.6 ± 20.5	81.5 ± 23.4	86.8 ± 23.1				
*8 week*	75.2 ± 17.7	83.1 ± 22.9	85.2 ± 26.0	88.6 ± 25.4				
*12 week*	75.1 ± 18.0	83.2 ± 19.3	82.9 ± 24.8	87.7 ± 24.2				
*16 week*	71.6 ± 18.1	84.4 ± 22.2	80.8 ± 22.9	83.9 ± 21.1				
*20 week*	74.7 ± 19.3	84.6 ± 20.7	83.4 ± 22.1	87.0 ± 22.6				
*24 week*	72.3 ± 17.3	82.5 ± 20.3	80.9 ± 22.5	83.5 ± 20.7				
								
**Serum Crt**, mg/dL (reference range: 0.5-1.3 mg/dL)					0.001	0.230	0.605	0.884
*Baseline*	0.80 ± 0.13	0.82 ± 0.11	0.78 ± 0.14	0.79 ± 0.09				
*4 week*	0.82 ± 0.17	0.80 ± 0.10	0.79 ± 0.13	0.79 ± 0.10				
*8 week*	0.80 ± 0.13	0.80 ± 0.12	0.80 ± 0.23	0.80 ± 0.11				
*12 week*	0.82 ± 0.14	0.81 ± 0.11	0.80 ± 0.14	0.80 ± 0.10				
*16 week*	0.81 ± 0.13	0.83 ± 0.10	0.79 ± 0.13	0.82 ± 0.10				
*20 week*	0.82 ± 0.14	0.82 ± 0.11	0.81 ± 0.14	0.80 ± 0.11				
*24 week*	0.79 ± 0.13	0.80 ± 0.11	0.77 ± 0.12	0.79 ± 0.09				
								
**Serum BUN**, mg/dL (reference range: 7-25 mg/dL)					0.178	0.158	0.118	0.433
*Baseline*	15.1 ± 3.1	15.8 ± 3.8	15.6 ± 4.1	15.6 ± 3.7				
*4 week*	16.7 ± 4.9	15.8 ± 3.4	16.4 ± 4.3	15.6 ± 4.1				
*8 week*	15.8 ± 3.8	14.7 ± 3.4	15.6 ± 3.7	15.6 ± 3.7				
*12 week*	16.4 ± 3.4	15.4 ± 3.9	15.8 ± 4.6	15.2 ± 3.5				
*16 week*	15.3 ± 3.6	15.9 ± 4.0	16.7 ± 3.6	15.9 ± 3.4				
*20 week*	15.9 ± 3.7	16.4 ± 3.7	15.2 ± 3.5	15.3 ± 3.4				
*24 week*	15.7 ± 3.7	15.7 ± 3.7	15.9 ± 5.1	15.3 ± 3.5				
								
**Serum Ca**, mg/dL (reference range: 8.6-10.2 mg/dL)					0.005	0.721	0.076	0.883
*Baseline*	9.4 ± 0.4	9.4 ± 0.3	9.4 ± 0.2	9.5 ± 0.3				
*4 week*	9.5 ± 0.4	9.4 ± 0.4	9.4 ± 0.3	9.4 ± 0.4				
*8 week*	9.5 ± 0.5	9.4 ± 0.3	9.4 ± 0.3	9.5 ± 0.4				
*12 week*	9.5 ± 0.4	9.4 ± 0.5	9.4 ± 0.3	9.4 ± 0.5				
*16 week*	9.4 ± 0.4	9.4 ± 0.3	9.5 ± 0.4	9.5 ± 0.4				
*20 week*	9.5 ± 0.4	9.3 ± 0.4	9.4 ± 0.3	9.5 ± 0.3				
*24 week*	9.4 ± 0.4	9.3 ± 0.3	9.3 ± 0.3	9.3 ± 0.4				
								
**Serum Pi**, mg/dL (reference range: 2.5-4.5 mg/dL)					0.001	0.547	0.027	0.825
*Baseline*	3.7 ± 0.5	3.6 ± 0.6	3.7 ± 0.5	3.7 ± 0.4				
*4 week*	3.8 ± 0.5	3.6 ± 0.5	3.7 ± 0.5	3.7 ± 0.5				
*8 week*	3.7 ± 0.5	3.6 ± 0.5	3.8 ± 0.5	3.8 ± 0.4				
*12 week*	4.0 ± 0.9	3.7 ± 0.5	3.8 ± 0.5	3.8 ± 0.4				
*16 week*	3.7 ± 0.5	3.7 ± 0.5	3.9 ± 0.5	3.8 ± 0.5				
*20 week*	3.8 ± 0.4	3.7 ± 0.5	3.8 ± 0.5	3.7 ± 0.5				
*24 week*	3.7 ± 0.4	3.6 ± 0.6	3.8 ± 0.5	3.7 ± 0.4				

GTP, green tea polyphenols; TC, Tai Chi; AST, aspartate aminotransferase; ALT, alanine aminotransferase; Bil, total bilirubin; ALP, alkaline phosphatase; Crt, creatinine; BUN, blood urea nitrogen; Ca, calcium; Pi, inorganic phosphorus.

Four participants reported side/adverse effects during the study. One subject in the Placebo arm experienced nausea and diarrhea several times. One subject in the GTP arm had elevated AST and ALT levels, possibly due to concomitant medications for cold symptoms (Ibuprofen 400 mg daily for 9 days), lowering cholesterol (Lipitor 20 mg daily) and hypertension (Metoprolol 25 mg daily). After discontinuation of the medication for cold symptoms, this patient's serum AST and ALT fell back to the normal range. One subject in the Placebo + TC arm reported having retinal bleeding on a non-exercise day, probably due to her uncontrolled high blood pressure and blood glucose, along with a family history of retinal bleeding. Another subject in the Placebo + TC arm reported having a broken wrist on a non-exercise day, due to an accidental fall. These four reports, as judged by the safety monitoring team, were unlikely related to the study protocol.

No adverse event due to TC was observed or reported in this study. There were only sporadic complaints about muscle soreness during the first two weeks.

### Quality of life

Data demonstrating the effects of GTP and TC on quality of life, including all 8 domains, in postmenopausal osteopenic women are presented in Table 3. At baseline, there was no significant difference in any domain of quality of life among all 4 treatment groups. Throughout the course of the 24-week intervention, there was no statistically significant change in any domain with time in all treatment groups, except that scores for physical function decreased with time (*P *< 0.001). However, when taking into account the interaction between time and the two treatment factors (GTP and TC), scores for physical function were not statistically different. Compared to those in the non-TC (Placebo and GTP) groups, subjects in the TC (Placebo + TC and GTP + TC) groups showed significant improvement in their scores for role-emotional (*P *= 0.036) and mental health (*P *= 0.003) after the 24-week intervention (Table 3). There was no significant difference in other domains of quality of life, including role-physical, bodily pain, general health, vitality, and social function (*P *> 0.05) (Table 3).

**Table 3:** Effect of green tea polyphenols supplementation and Tai Chi exercise on quality of life in postmenopausal osteopenic women

	Treatment groups				*P *(ANOVA)			
		
Domain	Placebo	GTP	Placebo+TC	GTP+TC	Time	Time ± GTP	Time ± TC	Time ± GTP TC
**Physical function**					< 0.001	0.214	0.414	0.453
*Baseline*	77.7 ± 21.4	77.6 ± 21.7	78.4 ± 16.3	80.4 ± 19.1				
*12 week*	72.9 ± 28.8	81.8 ± 21.2	80.9 ± 15.7	84.1 ± 15.6				
*24 week*	65.9 ± 20.6	68.4 ± 20.4	68.1 ± 16.3	69.4 ± 16.4				
								
**Role-physical**					0.136	0.477	0.578	0.466
*Baseline*	80.0 ± 34.7	78.4 ± 30.1	81.8 ± 33.2	84.6 ± 28.2				
*12 week*	85.7 ± 28.6	83.8 ± 30.7	80.4 ± 36.4	86.8 ± 26.3				
*24 week*	80.7 ± 31.6	89.2 ± 23.9	85.1 ± 28.5	89.7 ± 24.7				
								
**Bodily pain**					0.703	0.679	0.947	0.791
*Baseline*	73.8 ± 19.2	73.2 ± 17.5	74.5 ± 17.3	76.6 ± 15.3				
*12 week*	73.4 ± 19.3	74.2 ± 18.1	74.9 ± 16.5	76.2 ± 14.9				
*24 week*	74.5 ± 18.4	73.9 ± 17.0	77.3 ± 16.4	76.1 ± 17.2				
								
**General health**					0.168	0.676	0.917	0.114
*Baseline*	76.6 ± 16.5	72.9 ± 19.7	75.4 ± 16.2	76.4 ± 18.2				
*12 week*	77.8 ± 13.6	75.1 ± 21.4	77.1 ± 16.4	77.3 ± 16.9				
*24 week*	75.2 ± 16.0	76.0 ± 20.1	77.5 ± 16.2	76.5 ± 17.1				
								
**Vitality**					0.064	0.305	0.576	0.160
*Baseline*	63.0 ± 20.7	58.0 ± 24.1	60.8 ± 23.0	64.7 ± 18.9				
*12 week*	62.3 ± 22.4	59.5 ± 23.5	63.1 ± 21.7	67.9 ± 17.5				
*24 week*	60.6 ± 19.7	64.2 ± 22.7	65.4 ± 22.7	68.7 ± 19.1				
								
**Social function**					0.250	0.731	0.338	0.986
*Baseline*	89.3 ± 20.8	86.8 ± 20.2	85.1 ± 22.4	86.8 ± 16.6				
*12 week*	88.6 ± 20.2	88.2 ± 20.8	87.5 ± 22.8	91.9 ± 14.7				
*24 week*	85.7 ± 22.7	85.1 ± 22.4	86.1 ± 24.1	89.3 ± 18.2				
								
**Role-emotional**					0.464	0.918	0.036	0.815
*Baseline*	87.6 ± 29.2	88.3 ± 25.1	77.5 ± 36.9	81.4 ± 30.9				
*12 week*	89.5 ± 27.7	85.6 ± 31.0	81.1 ± 35.6	87.3 ± 30.7				
*24 week*	85.7 ± 32.6	82.9 ± 33.0	88.3 ± 26.3	91.2 ± 20.6				
								
**Mental health**					0.629	0.931	0.003	0.232
*Baseline*	80.2 ± 12.6	81.5 ± 11.5	77.4 ± 14.9	76.9 ± 15.7				
*12 week*	80.8 ± 14.4	78.7 ± 14.3	78.3 ± 16.9	82.0 ± 10.8				
*24 week*	78.6 ± 13.9	77.3 ± 17.1	81.3 ± 12.4	82.6 ± 10.0				

Data represent mean ± standard deviation (SD).

## Discussion

There is generally very little clinical information on the safety of long-term consumption of green tea extract supplements. The limited number of published studies were either short-term or with a small sample size, and most of them were not randomized controlled trials. This is the first placebo-controlled randomized study to evaluate the safety of long-term ingestion of green tea extract in postmenopausal women. This study demonstrated that supplementation of 500-mg GTP daily for 24 weeks did not cause any safety concern (Table 2) with regard to liver function (in terms of AST, ALT, Bil, and ALP levels) as well as kidney function (in terms of Crt and BUN levels).

Considering a typical commercial decaffeinated green tea bag that contains approximately 80-100 mg green tea flavanols per serving [22], the GTP daily dose (500 mg with 99.25% purity) used in this study was approximately equivalent to beverage prepared by 5-6 commercial decaffeinated tea bags. On the other hand, our previous animal study showed that GTP supplementation through 0.5% GTP in drinking water benefited bone remodeling in ovariectomized middle-aged rats [2]. This dose of GTP consumption by rats in that study was comparable to the dosage employed in the present study. GTP dosages similar to our study have been adopted in study populations with different health issues. However, the study periods were generally short (up to 12 weeks) in most studies with the following two exceptions. Matsuyama et al. [14] reported that 24 weeks of beverage ingestion containing catechin (576 mg daily) ameliorated serious obesity and cardiovascular disease risk factors without raising any safety concerns in obese Japanese children (aged 6-16 years). Janjua et al. [15] reported that GTP supplementation (500 mg with 70% catechin daily) for two years did not demonstrate a significant benefit superior to placebo in improving clinical or histological photoaging parameters of women's skin (aged 25 to 75 years). However, none of these studies investigated GTP's safety in terms of possible liver and kidney damages through monthly blood tests. Further, the sample sizes of these published studies were small.

In this study, we observed decreasing trends in the levels of serum Bil, ALP, Crt, Ca, and Pi over the study period (Table 2). However, such trends disappeared when analyzing interaction between the time factor and the two treatment factors (GTP and TC), suggesting possible body's adaptation to intervention stimuli over time.

In the present study, the four adverse events observed in different treatment arms were judged as unlikely related to the study protocol. Previous studies reporting adverse events with green tea extract supplementation, including acute liver failure in a few isolated case reports [23-26], in controlled human intervention trials [27,28], and in epidemiological studies suggested that possible medication contamination and other unknown factors may have contributed to hepatotoxicity [29]. Hepatotoxicity might also possibly be due to unusual dosing protocols, such as fasting, or a genetic variation (single nucleotide polymorphisms) in phase I and phase II enzymes in some affected individuals [30,31].

No adverse event attributed to TC was observed or reported in this study. This is in agreement with previous studies reported by us and others [16]. TC, featuring gentle, slow and flowing movements, has been considered a safe exercise with very low risk of injury. As expected, TC did not influence any parameters related to liver and kidney function, except for a decreasing trend of serum Pi with time, which became not significant considering interaction between time and TC (Table 2). In addition, there was no interaction between GTP supplementation and TC exercise on liver and kidney function in the present study.

The present results show that 24 weeks of TC exercise confers beneficial effects on postmenopausal women in terms of improving their role-emotional and mental health (Table 3). The favorable profiles of TC on mental health in the present study are consistent with those reported by Ko et al. [32] in healthy women, and by Abbott et al. [33] in patients with tension headaches. The positive impact of TC on the role-emotional domain also agrees with findings by Abbott et al. [33]. On the other hand, after involving GTP treatment, the interaction among time, GTP and TC was not significant (*P *> 0.05) in the domain of either role-emotional or mental health. Although time × TC did reach statistical significance, but time × GTP did not reach statistical significance, therefore, resulting in no significance in the results of time × GTP × TC.

This is the first study investigating the effect of GTP supplementation on quality of life, and the result showed no effect. There was also no evidence supporting that selenium supplementation benefited quality of life in apparently healthy elderly (aged 60-74) in a double-blind, placebo-controlled intervention [34]. Another study found that vitamin E intake did not change quality of life in patients with amyotrophic lateral sclerosis [35]. Although all these supplements (GTP, selenium, vitamin E) are considered to be functional in protecting cells from oxidative stress, these published studies along with the present study seem to suggest no benefit of these supplements in quality of life.

## Conclusion

Supplementation of 500-mg GTP daily to postmenopausal osteopenic women for 24 weeks did not cause any adverse effects on liver and kidney function, as determined by blood test parameters, and had no influence on quality of life (as assessed by SF-36 questionnaires). TC exercise for 24 weeks (3 hr/wk) significantly improved quality of life in terms of role-emotional and mental health in these subjects. Based on our findings, GTP at a dose of 500 mg per day and/or TC exercise at 3 hr/week for 24 weeks appear to be safe in postmenopausal osteopenic women.

## Competing interests

The authors declare that they have no competing interests.

## Authors' contributions

CLS received the research funding, led the entire study, and drafted the manuscript. MCC participated in the design of this study protocol and recruitment, implemented the exercise program, and drafted the manuscript. BCP, JKY, and JSW contributed to the design of this study protocol. CKF participated in the study design and oversaw participants' medical affairs. YZ participated in the design of the study and performed the statistical analysis. SD coordinated the study including blood/urine sample collection. All authors read and approved the final manuscript.

## Pre-publication history

The pre-publication history for this paper can be accessed here:

http://www.biomedcentral.com/1472-6882/10/76/prepub
